# Epilepsy, gut microbiota, and circadian rhythm

**DOI:** 10.3389/fneur.2023.1157358

**Published:** 2023-05-18

**Authors:** Yao Wang, Zhihong Zhuo, Huaili Wang

**Affiliations:** ^1^Department of Pediatrics, The First Affiliated Hospital of Zhengzhou University, Zhengzhou, China; ^2^Henan Provincial Key Laboratory of Childhood Epilepsy and Immunology, Zhengzhou, China; ^3^Henan Provincial Children's Neurological Disease Clinical Diagnosis and Treatment Center, Zhengzhou, China

**Keywords:** epilepsy, gut microbiota, circadian rhythm, microbiota–gut–brain axis, ketogenic diet

## Abstract

In recent years, relevant studies have found changes in gut microbiota (GM) in patients with epilepsy. In addition, impaired sleep and circadian patterns are common symptoms of epilepsy. Moreover, the types of seizures have a circadian rhythm. Numerous reports have indicated that the GM and its metabolites have circadian rhythms. This review will describe changes in the GM in clinical and animal studies under epilepsy and circadian rhythm disorder, respectively. The aim is to determine the commonalities and specificities of alterations in GM and their impact on disease occurrence in the context of epilepsy and circadian disruption. Although clinical studies are influenced by many factors, the results suggest that there are some commonalities in the changes of GM. Finally, we discuss the links among epilepsy, gut microbiome, and circadian rhythms, as well as future research that needs to be conducted.

## Introduction

Epilepsy is a chronic neurological disorder, which is a recurrent and transient brain dysfunction syndrome caused by abnormal synchronous firing of neurons (1). There are more than 65 million patients with epilepsy worldwide, with the majority coming from low-income countries (2). Due to seizures, mental disorders, cognitive deficits, and adverse drug reactions, epilepsy severely impacts patients’ daily life. Although great progress has been made in the diagnosis and treatment of epilepsy in recent decades, the specific mechanism of its onset and development still needed more studies. Current studies mainly focus on neuronal network reorganization, neuroinflammation, abnormal neurotransmitter release, axonal sprouting, and cell death (3, 4). Also, approximately 30% of epilepsy patients are resistant to conventional antiseizure medications (ASMs), therefore deep research on epilepsy becomes more essential (5, 6).

Recently, the microbiota–gut–brain (MGB) axis became more and more popular in neuroscience for its communication role among neuronal, endocrine, metabolic, and immunological pathways in both directions. The study has demonstrated that the intestinal flora plays a key role in central nervous system homeostasis, cognitive development, and behavior (7). Also, gut microbiota (GM) dysbiosis is associated with the onset and development of a number of neurological disorders, such as autism, multiple sclerosis, Parkinson’s disease, and Alzheimer’s disease. Alterations in the composition or function of GM were reported in epilepsy, particularly intractable epilepsy (8). Therefore, GM can become a target for drug treatment of epilepsy or a biomarker.

With the increased attention given to the circadian rhythm of epilepsy in recent years, time therapy has made great strides in the field of epilepsy treatment. However, to date only limited information on chronotherapy in patients with epilepsy is available. The circadian rhythm of seizures varies between different types of epilepsies (9). Choosing an appropriate medication timing according to the circadian rhythm can not only reduce the recurrence of seizures but also the compliance of patients, reduce adverse drug reaction, and improve the quality of life of patients (10, 11). Recently, the bidirectional relationship between GM and the circadian rhythm has also been extensively explored (12).

Limited pieces of literature focused on the relationship between GM, circadian rhythm, and epilepsy. In this review, we summarize the role of GM and the circadian rhythm in the development of epilepsy.

## Gut microbiota and epilepsy

### Gut microbiota

Gut microbiota is a complex microbial community composed of 10^8^–10^11^ cells of more than 1,000 different species that exert immune, metabolic, and protective functions through short-chain fatty acids, cytokines, and neurotransmitters (13). Bacterial communities, yeasts, fungi, protozoa, archaea, and viruses together maintain the balance of the ecosystem. Among them, *Firmicutes* and *Bacteroides* are the two most important phyla, followed by *Actinobacteria, Proteobacteria*, *Verrucomicrobia*, and *Fusobacterium* (14). The main function of the Bacteroides phylum is carbohydrate degradation, energy production and conversion, and amino acid transport. Their numbers decreased in intestinal microenvironmental disorders, obesity, and malnutrition. Unlike *Bacteroides*, *Firmicutes* increases in dysbiosis and obesity in mice. The abundance of *Proteobacteria* is very low in healthy people and upregulated in people with metabolic disorders or obesity. *Actinomycetes* is an anaerobic flora involved in maintaining homeostasis of the intestinal tract, constituted a small proportion of the GM, and declined with aging (15). The GM undergoes dynamic changes under the dual influence of internal (genetic) and external (nutrition, environment, infections, etc.) factors. Newborns mainly acquire actinomycetes and proteobacteria, which are less diverse from their mothers (16). Influenced by many factors such as prematurity, delivery methods, feeding methods, and antibiotic use, infants form a complex GM after 1 year of age, just like adults (17).

The GBM is a two-way information communication system that integrates the brain and gut functions. The diversity of GM is not only essential for gut health but also for the physiological function of other organs, especially the brain. There is an increase in epilepsy incidence in various gastrointestinal diseases. In turn, epilepsy affects the gastrointestinal tract in different ways (18). A cross-sectional study showed that irritable bowel syndrome (IBS) was more frequent in people with epilepsy compared with healthy controls, whereas IBS is associated with a greater burden of affective symptoms and insomnia (19). Another clinical study also indicated a significantly higher prevalence of functional gastrointestinal disorders in epileptic patients than in healthy individuals (20). These results suggest a bidirectional link between the gastrointestinal tract and epileptogenesis.

### Ketogenic diet (KD) and Gut microbiota

Among the factors influencing the components of GM, diet is the most important one. The diet causes changes in the microbiota, promotes the interaction of certain microorganisms, and causes differences in the concentration of neurotransmitters in the brain, which in turn affects seizures. The ketogenic diet (KD), which is based on high fat and low carbohydrate, causes a variety of changes in intermediate metabolism and resulted in ketone bodies as the major energy substrate, which is effective in focal and generalized seizures, pyruvate dehydrogenase deficiency, and glucose transporter-1 deficiency syndrome (21).

A recent study has shown that GMs are necessary and sufficient conditions for mediating the anticonvulsant effect of KD (22). After KD administration, not only the seizure threshold was increased but also the composition of GM was changed 4 days after diet treatment. KD-fed mice showed a decrease in alpha diversity, and the abundance of *Parabacteroides*, *Sutterella*, and *Erysipelotrichaceae* increased significantly. In antibiotic-treated and germ-free mice, KD did not increase the threshold of seizures, proving that the absence of microbiota has abrogated the effects of the KD. This suggests that the effect of KD on seizures is mediated by the GM. Zhang et al. (23) found that fecal microbial characteristics after KD treatment showed low alpha diversity, the frequency of *Firmicutes* was reduced, and the percentage of *Bacteroides* was increased. The researchers further analyzed the effect of KD on GM composition and function in patients with epilepsy and found that the relative abundance of *Bifidobacteria*, *Eubacterium rectale*, and *Dialister* was significantly reduced, while the relative abundance of *Escherichia coli* increased. Functional analysis revealed reductions in seven pathways including those involved in carbohydrate metabolism (24). These results may explain the corresponding proportional reduction in bifidobacteria and genes involved in carbohydrate metabolism during KD. Since the KD reduces the number of health-promoting, fiber-consuming bacteria, it also raises concerns about the overall health of the body (24).

### Animal model

Early animal model experiments have confirmed that GM mediates the development of behavioral symptoms and neuroinflammation in Parkinson’s disease (25), and is involved in the occurrence of autoimmune encephalomyelitis (26) and autism (27). In 2018, researchers demonstrated that transplanting GM from chronically stressed rats to young rats promoted the onset of epilepsy. This suggested that GM imbalance, especially under the influence of chronic stress, increased susceptibility to epilepsy (28). Francesca et al. (29) predicted that transplantation of the microbiota of epileptic mice may induce epileptic seizures by increasing brain excitability in healthy mice. Experimental results showed that mice that received microbiota derived from epileptic animals were more likely to develop status epilepticus than controls, suggesting that microbiota mediated seizure susceptibility. Further investigation of the relationship between gut inflammation and epilepsy revealed that gut inflammation increased seizure activity in epileptic mice. The authors believe that intestinal inflammation may be an effective target for epilepsy control and may also be a factor in seizures in susceptible patients (30).

Mahmoud Salami et al. (31) first investigated the effects of probiotic mixtures on pentylenetetrazol-triggered brain attack activity, cognitive performance, and concentrations of aminobutyric acid, nitric oxide, malondialdehyde, and total brain tissue antioxidant capacity in rats. The results showed that probiotics greatly reduced seizure severity. At the same time, oral administration of probiotics also partially improved spatial learning and memory in the kindled rats. Although inhibition/excitation of neurotransmission and an imbalance between antioxidants and oxidants are the main causes of seizures, treatment with probiotics increased GABA activity and improved the balance between antioxidants and oxidants in the kindled rats.

### Human clinical research

The possible role of gut microbes in epilepsy was first described in a case report. A 22-year-old patient with Crohn’s disease and a 17-year history of epilepsy was treated for Crohn’s disease with fecal microbiota transplantation (FMT). However, during the 20-month follow-up period, the patient no longer had seizures despite discontinuing treatment with sodium valproate (32). Researchers began to pay attention to the influence of GM on epilepsy. However, current human clinical research is mainly concerned with two aspects, one is the difference between the gut flora of epilepsy patients and healthy people, and the other is the improvement of symptoms of epilepsy patients after taking probiotics/FMT.

There have been several clinical studies on the differences in GM between epilepsy patients and healthy controls (Table 1) (33–39). Using 16S rDNA sequencing, Xie et al. (33) analyzed fecal microbiome sequences from 14 epileptic infants and 30 healthy infants. The results showed higher diversity and increased Bacteroides in healthy infants compared with infants with drug-resistant epilepsy (DRE). Similar results were obtained by Korean experts in 2021, showing a lower number of Bacteroides in the epilepsy group compared to the control group (34). In 2018, Peng et al. (35) used the same technology to compare the stool microbiota of 49 drug-sensitive epilepsy patients (DSE), 42 DRE patients, and 65 healthy controls. Compared with the DSE group and the control group, the DRE patients showed increased alpha diversity, increased *Firmicutes*, and decreased *Bacteroidetes*. In contrast, many other rare phyla showed an increased trend in the DRE group, such as Verrucomicrobia. Lee et al. (36) also analyzed the difference in GM between DRE and DSE. In the DSE group, the relative abundance of *Bacteroides finegoldii* and *Ruminococcus_g2* increased, while in the DRE group, the relative abundance of Negativicutes, which belong to *Firmicutes,* increased. In another study, a total of 55 epilepsy patients and 46 healthy controls (spouses) were recruited and their stool samples were collected for 16S rRNA sequencing and microbiological analysis. The results showed changes in the gut microbiome of the epilepsy group, including a decrease in alpha diversity, an increase in *Actinobacteria* and *Verrucomicrobia*, the decrease in *Proteobacteria* at the phylum level, and an increase of *Prevotella_9*, *Blautia*, *Bifidobacterium*, and others at the genus level (37). However, Birol Şafak et al. (38) found that *Proteobacteria* and *Fusobacteria* in epilepsy patients were higher than those of the healthy group, and related reports indicated that *Proteobacteria* and *Fusobacteria* were increased in autoimmune diseases and inflammatory bowel diseases (40). This suggests that autoimmune mechanisms and inflammation may play a role in the etiology of epilepsy. A recent study also found an enrichment of *Proteobacteria* in drug-naive epileptic patients (39). Clinical studies have pointed out the differences in GM between DRE patients, DSE patients, and healthy controls, suggesting that differential GM can be used as biomarkers for predicting prognosis and evaluating treatment response in epilepsy patients. However, most participants in previous studies were enrolled after taking ASMs, it is possible that drug confounding may affect the differences in GM. Recently Ilhan et al. (41) reported that ASMs affect the growth of gut bacterial species and subsequent host response. The first-generation carbamazepine and the second-generation lamotrigine have greater antimicrobial activity than other ASMs. This may be related to the different efficacy that ASMs have on seizure control. Therefore, in subsequent clinical studies, we should pay attention to exclude potential confounding factors such as age, diet, and ASMs to ensure the accuracy of future experiments.

**Table 1:** Summary of previous studies on the gut microbiota in patients with intractable epilepsy.

References	Region	Study Cohort	Age	Result
Xie et al. (33)	China	Refractory epilepsy (*n* = 14) and HC (*n* = 30)	EP: 1.95 ± 3.10 HC: aged up to 3 years	HC: higher GM diversity； more than twice *Bacteroides* than infants with refractory epilepsy EP: Phylum: increases in Firmicutes and Proteobacteria Genus: increases in *Cronobacter*
Lee et al. (34)	Korea	Intractable epilepsy (*n* = 8) and HC (*n* = 32)	EP: 3.49 ± 1.76	EP: increases in Actinobacteria; decreases in Bacteroidetes and microbiota richness indices
Peng et al. (35)	China	DRE (*n* = 42), DSE (*n* = 49), HC (*n* = 65)	DRE: 28.4 ± 12.4 DSE: 5.1 ± 14.6 HC: 29.4 ± 13.8	DRE: increases in Firmicutes and Verrucomicrobia; decrease in Bacteroidetes
Lee et al. (36)	Korea	DRE (*n* = 23) and DSE (*n* = 21)	DRE: 41 ± 13.6 DSE: 44 ± 17.2	DSE: increases in *Bacteroides finegoldii and Ruminococcus_g2* DRE: increases in *Negativicutes of Firmicutes* epilepsy patients with a normal electroencephalogram: increases in *Bifidobacterium*
Gong et al. (37)	China	Exploration Cohort (EP *n* = 55 and HC *n* = 46) and Validation Cohort (EP *n* = 13 and HC *n* = 10)	EP: 26.33 ± 12.05 HC: 28.5 ± 4.27	EP: lower alpha diversity; Phylum: increases in Actinobacteria and Verrucomicrobia and decreases in Proteobacteria; Genus: increases in *Prevotella_9, Blautia, Bifidobacterium* DRE: Phylum: increases in Actinobacteria, Verrucomicrobia, and Nitrospirae Genus: increases in *Blautia, Bifidobacterium, Subdoligranulum, Dialister, and Anaerostipes*
Şafak et al. (38)	Turkey	Idiopathic focal epilepsy (*n* = 30) and HC (*n* = 10)	EP: 41.3 ± 12.2 HC: 1.7 ± 6.8	EP: increases in Proteobacteria (*Campylobacter, Delftia, Haemophilus, Lautropia, Neisseria*) and Fusobacteria
Ceccarani et al. (39)	Italy	Drug-naive children (*n* = 8) and HC (*n* = 7)	EP (epilepsy onset): 8.9 ± 4.3 HC: 8.0 ± 4.2	EP: increases in *Akkermansia* spp. and Proteobacteria; decreases in *Faecalibacterium* spp.

DRE, drug-resistant epilepsy; DSE, drug-sensitive epilepsy; EP, patients with epilepsy; HC, health control group. Patient’s age was expressed as the mean ± SD.

Considering the MGB axis, dietary supplements (probiotics and prebiotics) may be a useful measure for the treatment of epilepsy. A prospective study investigated the association between neonatal seizures and rotavirus infection and identified significant factors that may be related to the pattern of white matter injury (WMI) in seizures and rotavirus infection (42). The results suggest that rotavirus infection is an independent risk factor for neonatal seizures and is associated with WMI. Probiotics administered immediately after birth can reduce rotavirus-related neonatal seizures by 10-fold. Researchers believe that probiotics may reduce the risk of rotavirus-associated neonatal seizures through the inhibition of non-structural protein 4 or through antiinflammatory effects (42–44). A pilot, open-label, single-center, and prospective clinical study investigated the effects of probiotics as adjunctive antiepileptic therapy on epilepsy control and quality of life (QoL) in patients with DRE (45). After 4-month use of probiotics in 45 patients with DRE, seizures decreased by 50% in 28.9% of patients, and the QOLIE-10 test showed that patients’ QoL was significantly improved with effective probiotics. The abovementioned research findings suggest that probiotic supplementation in DRE is safe, reduces seizure frequency, and improves the quality of life.

These studies suggest an inextricable link between gut microbiota and epilepsy, especially intractable epilepsy. Probiotic intervention and fecal microbiota transplantation can effectively improve seizures and quality of life.

## Epilepsy and circadian rhythm

### Time window of epileptic seizures

According to the findings of electrophysiology, seizures in patients with epilepsy are more likely to occur at a specific time in the circadian cycle, referred to here as peak seizure time. The different peak time of seizures in each subtype of epilepsy also suggests that seizure types have a circadian rhythm. Evidence suggests that myoclonus, atonic seizures, and epileptic spasms occur more frequently during wakefulness, and tonic, clonic, and hypermotor seizures occur more frequently during sleep (46). A retrospective on 407 children with epilepsy relied on video-electroencephalography results to understand the development of generalized tonic–clonic seizures (GTC). The results show that the development of GTC occurs most frequently in the early morning, especially in patients with extratemporal epilepsy and in patients without MRI injury. Focal epilepsy is found to be more likely to occur outside of sleep, opening a new direction for our timed treatment of epilepsy management (47).

In addition to the differences in the localization of the epileptic lesions, there are also differences in the peak times of the seizures. Intracranial seizure-like activity rhythms were monitored in patients with focal epilepsy for 84 consecutive days using the RNS system and showed a strong 24-h periodicity with a peak at night. Limbic and temporal lobe epilepsies exhibited different circadian rhythms, suggesting that the circadian rhythm pattern of epileptiform activity varies depending on the onset area (48). Previous studies have reported that frontal lobe seizures occur mostly at night and during sleep (46, 49), whereas temporal and occipitoparietal lobe seizures occur more frequently during wakefulness (49, 50). A retrospective study also indicated that temporal lobe seizures occurred during waking hours (06:00–09:00 and 12:00–15:00), occipital seizures occurred during daytime and waking hours (09:00–12:00 and 15:00–18:00), and parietal seizures also occurred mainly during daytime (46). Fukuda et al. (51) compared the circadian rhythm characteristics of patients with juvenile myoclonic epilepsy (JME) and patients with temporal lobe epilepsy (TLE). The results showed that JME patients had no obvious circadian rhythm pattern compared with TLE patients, but most patients with JME are in poor condition in the morning.

The relationship between the clinical features of children with postencephalitis epilepsy and the diurnal rhythm of seizures was summarized in an article by Li et al. (11). Regarding seizure types, tonic seizures mostly occur at night and during sleep; epileptic spasms mostly occur during daytime and awake (11). The author compared gender differences and found that seizures occurred more frequently during wakeful periods in boys than during sleep periods in girls (11). This suggests that hormones may play a role in the circadian rhythm of epilepsy. As for prognosis, epileptic spasms are more likely to occur in the waking state, and the prognosis is relatively worse (11). This finding brings us to the origin of temporal lobe epilepsy, which occurs most frequently during wakefulness and is also known as the most common form of refractory epilepsy. Sudden unexpected death in epilepsy (SUDEP) is the leading cause of premature death in patients with refractory epilepsy. SUDEP usually occurs at night, but the specific mechanism is unclear. Purnell’s team used two mouse models for seizure-related death, DBA/1 mice and C57BL/6 J mice. In DBA/1 mice with normal locomotion, the time of day can alter the likelihood of seizure-related death. In free-running C57BL/6 J mice that elicit maximal electroshock seizures at the same time at different circadian times, the circadian phase may alter the probability of seizure-related death. In both mouse models, the probability of seizure-related death is greatest at night. The abovementioned results suggest that circadian rhythm may be the reason for the increased nocturnal prevalence of SUDEP (52) Earlier studies by Kalume et al. found that the circadian rhythm of mice with Dravet syndrome (DS) exhibited behavioral defects, including decreased circadian rhythm amplitude of wheel running activity (WRA), prolonged endogenous WRA cycles in constant darkness, and a prolonged time for WRA to retrain a new light–dark cycle (53). Later, the research team examined circadian sleep regulation in DS mice. The results showed that sleep regulation was disrupted, including fragmentation of the sleep rhythm without rapid eye movements and prolongation of the circadian sleep cycle (54).

### Connection between epilepsy and circadian rhythm

Circadian rhythm refers to the sleep–wake rhythm, physiological and psychological behavior, and biology under the control of the biological clock, including changes in sleep and wakefulness, core body temperature, blood pressure, and hormone levels. Disruption of the rhythm has negative effects on human health.

### Animal model

In animal studies, epilepsy models induced by electrical stimulation or excitatory drugs have shown seizures regulated by the circadian rhythm (55, 56). However, it is controversial whether the frequency of seizures in rats induced by pilocarpine is related to the circadian rhythm (57). In 2017, a study that also used pilocarpine to induce epileptic mice showed that the circadian rhythm of seizures in status epilepticus (SE) occurred after 4 days (58). In 2019, Baud et al. (59) also examined the epileptic activity cycle of mice at different periods after status epilepticus (chronic phase and incubation period) and at different stages of brain injury and also found that they would occur early after SE. Gregg et al. (60) established an epilepsy model using dogs as research subjects and assessed the aggregation of seizures using the dispersion index. The results showed that the timing of seizures in dogs was not random and that circadian and multiday periodic seizures and seizure clusters were common. Moreover, circadian rhythm and multiday seizures were not associated with ASMs dose, and these patterns may reflect the endogenous rhythm of seizure risk.

### Human clinical research

A retrospective cohort study using data from the two most comprehensive human seizure databases included 12 patients with refractory epilepsy from the NeuroVista database and 1,118 patients with Seizure Tracker. Results showed that at least 891 (80%) of 1,118 patients and at least 11 (92%) of 12 NeuroVista patients showed circadian rhythm regulation of seizure rates (61). Campen and colleagues used published data to visually compare the circadian rhythms of epileptic seizures with those of cortisol, which are similar, especially when seizures increase in the early morning hours and subside at night. This similarity can be observed in both children and adults, but there are differences between the different seizure types and the location of the epileptic foci (62). Karoly’s team developed a specific epilepsy prediction model based on the circadian rhythm of epilepsy. It is encouraging that implantable devices that can continuously record and store neuronal data have been developed to apply probabilistic epilepsy prediction in clinical practice (63). To investigate the relationship between seizure timing and fluctuations in interictal epileptiform activity (IEA), the team enrolled 37 epilepsy patients implanted with brain stimulation devices. IEA fluctuated with circadian rhythms and multiple cycles over several years, further improving the ability to predict seizure risk. It is possible to provide dynamic and personalized treatment strategies (9).

### Role of core clock genes in epilepsy

Circadian rhythms in mammals are regulated by master clock genes and peripheral organ clock genes located in the suprachiasmatic nucleus (SCN) of the hypothalamus (64). CLOCK and BMAL1, the core of the circadian system molecule, form a heterodimer complex in the cytoplasm that is phosphorylated by a protein kinase and migrates to the nucleus, combines with the E-box sequence in DNA, and regulates the transcription of related genes. These genes form a negative feedback pathway, inhibit the transcription of clock genes, create shocks at the gene level, and thus lead to a circadian rhythm (65). There are two pathways of negative feedback. First, PER-CRY complexes produced by Per (Per1 and Per2) and Cry (Cry1 and Cry2) genes bind to CLOCK /BMAL1 complexes to inhibit their transcription. Second, CLOCK /BMAL1 binds to the E-box sequence to activate the PAR bZIP transcription factor, which consists of the Dbp, Tef, and Hlf genes. The abovementioned E-box sequence is controlled by the CLOCK /BMAL1 complex, whereas the D-box sequence is regulated by both PAR bZIP and Nifl3, with Nfil3 being the transcriptional suppressor of the D-box sequence. Activation of the transcription factors REV-ERB α/β and RORα/β requires the binding of CLOCK/BMAL1 to the D-box and E-box and D-box sequences, respectively, and they share a common binding site-the orphan receptor response element (RORE). The Ror protein can act on the transcription of the circadian rhythm genes Bmal1 and Nifl3 activated by RORE, while the REV-ERB protein plays an inhibitory role. Therefore, the PAR bZIP protein and Nifl3, which regulate the Rev. and Ror genes, indirectly inhibit transcription of the Bmal1 gene (66, 67).

The master clock regulates the release of neurotransmitters, such as serotonin and norepinephrine. Serotonin and norepinephrine are at high levels during the day, while melatonin peaks at night (68). There is evidence that serotonin has a protective effect on neuronal death caused by epilepsy (69). A recent study showed that melatonin significantly increased the expression of circadian rhythm genes and ameliorated NMDA-induced seizures. It has been suggested that the anticonvulsant effect of melatonin may be related to the regulation of circadian rhythm gene expression (70). Felix Chan et al. (66) summarized the potential downstream pathways of the circadian molecular system linking circadian rhythm and epilepsy, namely regulation of pyridoxal metabolism, mammalian target of rapamycin (mTORC) signaling, and redox state, and for the upstream factors affecting circadian rhythm genes, we need to consider GM. Using the Kcna1 knockout, an epileptic mouse model was constructed, and the Clock, Per1, and Per2 genes fluctuated significantly in wild-type (WT) and epileptic mice under the “artificial day and night” environment of 12 h of care and 12 h of darkness, indicating the influencing factors of time on Clock gene expression. Compared with the mice from WT, the total mRNA expression of Clock, Per1, and Per2 was decreased in epileptic mice (71). The researchers also analyzed clock gene expression in WT and epileptic mice exposed to continuous darkness and found that only Per2 expression was affected, suggesting that the circadian rhythm of epileptic seizures may be regulated by endogenous circadian rhythms. This study examined the temporal expression and spontaneous movement activity (SLA) of seven key circadian transcripts (Bmal1, Clock, Cry1, Cry2, Per1, Per2, and PER3) in a post-status epilepticus (SE) model of mTLE. The 24-h oscillation SLA remained intact in the post-SE group. However, in the early post-SE and epileptic phase, circadian rhythms and activity volume and intensity changed. After SE, all clock transcripts except Per2 and Per3 were significantly dysregulated (72).

Decreased Clock protein level was observed in epileptic tissue samples from patients with focal epilepsy, and deletion of the Clock gene reduced seizure threshold in mice (73). Rev-ERBA expression is downregulated in the epileptic region of patients with TLE. Rev-erba inhibitors inhibit NLRP3 inflammasome activation, inflammatory cytokine production (IL-1β, IL-18, IL-6, and TNFα), astrocyte proliferation, microglial hyperplasia, and hippocampal neuronal damage according to SE. These results suggest that the reduction of Rev-erba in the epileptic region may be involved in the TLE process, and that activation of Rev-erba may have antiinflammatory and neuroprotective effects (74).

### Role of the mammalian target of rapamycin (mTOR) in the circadian rhythm of seizures

Mammalian target of rapamycin (mTOR) is a serine/threonine protein kinase related to cell growth and proliferation, which regulates cell growth and metabolism, affects transcription and protein synthesis, and regulates cell apoptosis, autophagy, etc. (75). It is known that abnormal activation of the mTOR pathway can cause a number of neurological diseases such as tuberous sclerosis and epilepsy (76). In the hypothalamus, mTOR acts as a metabolic sensor to control food intake and regulate energy balance (77). The activity of the mTOR pathway in the SCN has been shown to be strong under light control, suggesting that mTOR signaling plays a role in regulating the circadian clock (78). MAPK, the upstream signaling pathway of mTOR, mediates light activation of mTOR (78). In addition, the PTEN-Akt-RHEb-TOR-S6K pathway has also been found to influence the circadian cycle in Drosophila (79). A number of key factors in the mTOR pathway are regulated by biological clock genes (80). Conversely, key factors in the mTOR pathway also regulate biological clock genes. mTOR regulates protein production and phosphorylation of the central clock nucleus BMAL1, thereby controlling BMAL1 levels and affecting its translation, degradation, and subcellular localization (81). Thus, activation of mTOR signaling pathways can alter the CLOCK gene BMAL1–CLOCK complex and downstream transcription factor function, leading to seizures and changes in circadian rhythm.

To sum up, epileptic seizure type have circadian rhythm. The core clock genes CLOCK and BMAL1 play important roles in epilepsy. In addition, the mTOR signaling pathway may act as a bridge between seizures and circadian rhythm changes.

## Gut microbiota and circadian rhythm

### Circadian rhythm of Gut microbiota activity

In this age of fast-paced life, circadian rhythm disruption affects most people. Research in recent years has also shown that circadian rhythm disruption increases the likelihood of a variety of diseases, including obesity, diabetes, cardiovascular disease, cancer, and neurodegenerative diseases (82–85). Gut microecology is a hotspot of research in the new era and mediates a variety of chronic, inflammation-related diseases. In addition, the question of whether there is circadian activity has attracted the attention of scientists as the “second largest gene pool” in humans. The results of the study are impressive: approximately 60% of the composition of the gut microbiome exhibits a circadian rhythm, more than 20% of the symbiotic bacterial species of the gut show significant diurnal variation in mice, and 10% of the symbiotic species of humans also exhibit diurnal variation (86). The mechanism of interaction between GM and circadian rhythms is not entirely clear. However, recent studies reported that GM regulates the circadian rhythm of host metabolism via histone deacetylase 3 (HDAC3). The specific content is that the microbiota induces the expression of HDAC3 in intestinal epithelial cells and HDAC3 is rhythmically recruited to chromatin and generates synchronized circadian oscillations in histone acetylation, metabolic gene expression, and nutrient uptake (87). The circadian rhythm of the intestinal flora is closely related to microbiota composition and function and influences host immunity and metabolism. Therefore, related scientists have suggested that microbiology-based therapy could improve the imbalance caused by circadian rhythm disturbances (12).

### Changes in circadian rhythms cause dysregulation of intestinal microbial

As a control center of circadian rhythm, the SCN is mainly influenced by light factors (88). When external light conditions change, circadian rhythms are disrupted. Researchers have also studied this extensively. When mice were housed in constant darkness, their circadian rhythms were disrupted and the gut flora changed accordingly. It was found that bacterial diversity decreased, Firmicutes increased and Bacteroides decreased. Similarly, disruption of the host circadian rhythm also alters the gut microbial community and functional gene composition when switching to continuous illumination conditions (12). Voigt et al. (89) reported that the composition of GM was altered in male C57BL/6 J mice with disrupted circadian rhythms compared with controls. In addition, we know that peripheral biological clocks also play an important role in regulating circadian rhythms. When the normal sleep rhythm changes, such as during shift work and chronic jet lag, the circadian rhythm of the host GM also changes due to the transcriptional oscillation of peripheral clock genes and the change in feeding time pattern (90). To create a jet lag model, mice were exposed to an 8-h time shift every 3 days. After 4 weeks of jet lag induction, the host lost rhythmic activity. Subsequent microbiome analysis of mice in the experimental group every 6 h showed that the bacterial rhythm disappeared and the number of oscillations of the operational classification units decreased. Moreover, as the time caused by jet lag increases, the extent of intestinal dysbiosis also increases (86). Disruption of GM caused by jet lag may promote impaired glucose tolerance and obesity. Interestingly, the germ-free mice without jet lag induction exhibited metabolic abnormalities when the feces of the experimental group were transplanted into the germ-free mice. This suggests that the gut flora is involved in the metabolic abnormalities caused by circadian rhythm disruption (86).

Earlier we talked about the central clock genes CLOCK and BMAL1 for circadian rhythm. Does the mutation or deletion of the core clock genes affect the imbalance of the gut flora? We will talk about this in detail below. In an experimental setup, a clock mouse model was constructed and different diets were administered, a standard diet, an alcoholic diet, and an alcohol control diet with glucose instead of alcohol calories. The results show that compared to wild-type mice, the diversity of the microbial community in the gut of the clock gene mutants is reduced and the imbalance of the gut flora is exacerbated under the alcohol-containing diet (91). Similarly, the symbiotic bacterial diversity in the Per1/2−/− mouse model almost completely lost the rhythmic fluctuations. To determine whether the disappearance of the rhythm of microbial flora affected the activity of metagenomic metabolic pathways, the researchers performed shotgun sequencing of metagenomic DNA in Per1/2−/− and WT mice. In Per1/2−/− mice, pathways involved in vitamin metabolism, nucleotide metabolism, secretion system, DNA repair, cell wall synthesis, and the movement lost their daily rhythm. The abovementioned results suggest that the daily fluctuation of gut flora composition and function requires the host biological clock (86).

### Dysregulation of intestinal microbial causes fluctuations in circadian rhythm expression

The gut microbiome is a key factor influencing the development and function of the central nervous system. As mentioned earlier, the microbiome–gut–brain axis is a hot research topic and also an urgent topic to discuss. According to the study, GM is a key factor in regulating circadian rhythms. Germ-free mice fed a low-fat or high-fat diet showed significantly impaired expression of central circadian clock genes when exposed to light/darkness for 12:12 h and dietary conditions were changed (92). Weger et al. (93) constructed germ-free and antibiotic-treated mouse models and found that intestinal dysbiosis can also alter the expression of peripheral and intestinal clock genes. The research findings of Mukherji’s team are consistent with the above findings (94). In addition, the elimination of gut microflora can alter the expression of Rev-erba and RORα in the gut (95). However, it has also been reported that the elimination of gut microflora by antibiotics is not associated with circadian rhythm oscillation in mice (96). The reason for the different results could be due to the different assay methods or sample preservation methods.

The GM regulates the circadian clock and is influenced by exogenous factors such as diet and mealtime (92). We have talked about clock gene expression being affected in response to changes in diet. Researchers have proposed a new dietary pattern. Intermittent fasting has been shown to improve many chronic diseases, such as obesity, hypertension, and hyperlipidemia (97, 98). Ye Yuqian et al. (99) categorized the mice in the experimental study into three groups, namely the free diet group, the high-fat diet group, and the high-fat diet group, which was restricted within 8 h. The results showed that mice on the time-restricted high-fat diet gained less weight than mice on the free high-fat diet, and there were differences in the numbers of Bacteroidetes and Firmicutes between the two groups. Compared with mice on the normal diet, the circadian rhythms of SIRT1, SREBP, and PPAR expression were more distinct in the livers of mice on the time-restricted high-fat diet and the abundance of Bacteroidetes and Firmicutes. The researchers suggest that the eating–fasting rhythm may stimulate fluctuations in our GM and subsequent molecular changes that restore a healthier body clock (100).

Metabolites of intestinal flora, such as SCFAs and unbound cholic acids, may also affect the expression of circadian genes. SCFAs also show rhythmic changes throughout the day, and detection of SCFAs in feces shows that the concentration of SCFAs is highest in the morning and gradually decreases (101). SCFAs play a positive role in protecting the integrity of the intestinal barrier (102). In addition, the reduction of SCFA-producing bacteria may result in the release of proinflammatory bacterial products into the systemic circulation, which triggers and promotes inflammatory diseases (103). Therefore, SCFAs have a beneficial protective effect on a variety of diseases (104). Leone et al. (92) set up a liver cell model, and after administration of butyrate or a small amount of acetate, the expression of the Per2 and Bmal1 genes was significantly increased. The next step was to determine whether short-chain fatty acids directly affect the host clock *in vivo*. In germ-free mice, butyrate treatment significantly increased the proportion of Per2:Bmal1 mRNA in liver cells 2 h after illumination (the active period of the mice) for 5 days, whereas the same treatment did not result in a significant increase in the proportion of Per2:Bmal1 mRNA in the mediobasal hypothalamus (92). The experimental results further confirm the author’s conjecture. Govindarajan et al. (105) use a synchronized Caco-2 epithelial cell model to demonstrate that unbound bile acids can improve the level of circadian rhythm gene expression. Further oral administration of unbound cholic acid to mice surprisingly showed significant changes in circadian gene expression levels in the ileum, the colon, and the liver.

These data suggest that GM has a potential impact on circadian rhythm expression. An in-depth study of the interaction between gut microbiota and circadian rhythm can help in better understanding the regulation of host physiological functions by gut microbiota.

## Relationship between epilepsy, Gut microbiota, and circadian rhythm

To date, most studies of the influence of GM or the circadian rhythm on disease have been conducted independently and have not combined GM and the circadian rhythm to examine the joint effects of the two. When the circadian rhythm is disrupted, it can affect host immunity and metabolism through the gut flora and promote the onset of disease. Conversely, the imbalance of the GM can affect the circadian rhythm. When the steady state of the circadian rhythm is disrupted, it further affects host homeostasis. Many publications have investigated the relationship between epilepsy and GM, and the circadian rhythm of GM has been gradually recognized, but no hypothesis has yet been proposed for the relationship between GM, the circadian rhythm, and epilepsy.

**Figure 1:**
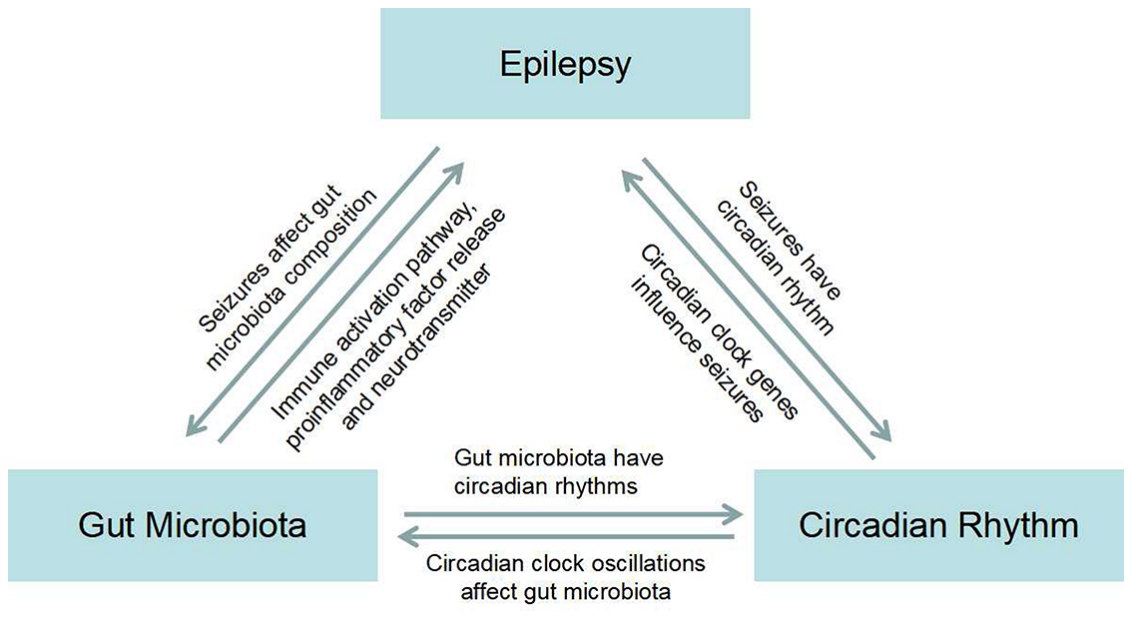
Map of the bidirectional association between epilepsy, gut microbiota, and circadian rhythms.

GM influences functional brain signaling through the MGB axis, and brain signaling also influences the activities and physiological functions of long-lived microorganisms (106). GM can be mediated through the pathway of immune activation, proinflammatory factor release, and neurotransmitter release, causing epilepsy. Seizures may in turn cause dysbiosis in the gut. There is a bidirectional relationship between GM and the occurrence of epilepsy (107). Different types of seizures and the origin of lesions have different peak seizure times, suggesting that seizures have a circadian rhythm (108). Circadian clock genes regulate the occurrence and development of epilepsy (9). Similarly, there is a bidirectional signal between the two. Next, we will talk about GM and circadian rhythms, which, not surprisingly, also have the ability to transmit signals in both directions (12). Previously, the three formed a bidirectional link between two signaling pathways (Figure 1). In our literature search, we found that Firmicutes increased and Bacteroidetes decreased in epilepsy patients compared with healthy control subjects. Surprisingly, under the condition that the circadian rhythm changes, the bacteria that alter the intestinal flora also showed an increase in Firmicutes and a decrease in Bacteroidetes. This led us to ask whether the interaction between GM and circadian rhythms might play a role in seizures. Therefore, we next need to explore whether there is a circadian rhythm in the GM of patients with refractory epilepsy and the mechanism by which the interaction between circadian rhythm and GM plays a role in epilepsy. The mechanism by which the gut flora mediates the anticonvulsant effect of KD has already been mentioned. We know that KD is a high-fat, low-carbohydrate diet, which reminds us that the expression of circadian clock genes is also influenced by diet, such as low-fat and high-fat diets. Whether clock genes are involved in GM-mediated anticonvulsant effects of KD is the next question we need to ask.

## Limitations of the review and future research

In this review, we tried to describe the mutual relationship between GM, circadian rhythm, and epilepsy. Unfortunately, the published literature has studied the interaction between them independently, so in terms of the mechanism of interaction between epilepsy, GM, and circadian rhythm, many unknown problems have occurred. GM is susceptible to the influence of age, diet, drugs, DNA sequencing, and other factors, resulting in different research results. We suggest that these variables should be more strictly controlled in future clinical studies, and the changes in intestinal microbiota at the species and strain levels should be further clarified. Functional analysis and association analysis combined with metabolomics should be carried out to better reveal the possible mechanism between GM dysbiosis and diseases. In addition, more studies are needed to explore the specific mechanisms of how circadian rhythm disorder leads to GM dysbiosis, and how the synergistic effect of circadian rhythm disorder and GM can trigger the process of epilepsy.

## Conclusion

With the advancement of sequencing technology, the inextricable links between GM and epilepsy have been gradually revealed, leading to a deeper understanding of their functions. By exploring the relationship between GM and epilepsy, we may discover sensitive biomarkers that improve the understanding of the complex mechanisms of epilepsy. Currently, some clinical studies have confirmed that there are differences in gut microbiota between patients with refractory epilepsy, drug-sensitive patients, and healthy controls. At the same time, GM also mediates the pathogenesis of KD in the treatment of refractory epilepsy. Overall, future microbiome-specific treatment may be an effective option for refractory epilepsy. The circadian rhythm of GM seems to play an important role in the onset and development of epilepsy and also points the direction of our next research. Discovering the relationship between GM, circadian rhythm, and epilepsy will help us to better understand the pathogenesis of epilepsy and thus improve the quality of life of patients with epilepsy.

## Author contributions

YW was responsible for the execution of the research project and writing of the manuscript. ZZ assisted in writing the manuscript. HW conceptualized and designed the study and reviewed and revised the manuscript. All authors read and approved the final manuscript.

## Funding

This study was supported by the Natural Science Foundation of Henan Province, No. 202300410469 and the Henan Provincial Key Project of Science and Technology Research, No. SBGJ2020002054 and SBGJ202102109.

## Conflict of interest

The authors declare that the research was conducted in the absence of any commercial or financial relationships that could be construed as a potential conflict of interest.

## Publisher’s note

All claims expressed in this article are solely those of the authors and do not necessarily represent those of their affiliated organizations, or those of the publisher, the editors and the reviewers. Any product that may be evaluated in this article, or claim that may be made by its manufacturer, is not guaranteed or endorsed by the publisher.
